# Improved MeSH analysis software tools for farm animals

**DOI:** 10.1111/age.13159

**Published:** 2021-12-02

**Authors:** Sabrina T. Amorim, Koki Tsuyuzaki, Itoshi Nikaido, Gota Morota

**Affiliations:** ^1^ Department of Animal and Poultry Sciences Virginia Polytechnic Institute and State University Blacksburg VA 24061 USA; ^2^ Laboratory for Bioinformatics Research RIKEN Center for Biosystems Dynamics Research 2‐1 Hirosawa Wako Saitama 351‐0198 Japan

## Background

The Medical Subject Headings, also known as MeSH, comprise a controlled life sciences vocabulary maintained by the National Library of Medicine for indexing journal articles. These headings are manually curated at the National Center for Biotechnology Information to be used in the MEDLINE database accessible from PubMed. Mapping MeSH IDs to Entrez Gene IDs can turn it into a powerful resource for enrichment analysis. We developed MeSH annotation Bioconductor packages for over 80 species coupled with a MeSH enrichment analysis package that initially became available in April 2014 as Bioconductor 2.14.^1^ We reported the first detailed MeSH enrichment analysis of cattle, swine^2^ and chicken^3^ accompanied by reproducible R code for MeSH analysis. A unique aspect of MeSH is that both the quantity and quality of annotations are driven by new knowledge that is disseminated via peer‐reviewed scientific articles.^1^ It may therefore be viewed as a community‐driven annotation project that is expected to improve over time with the publication of more scientific articles. In October 2021, the MeSH framework underwent a major change starting from Bioconductor 3.14 that resulted in the previous R code becoming dysfunctional. The objective of this study was to report improvements in MeSH annotations since its release in 2014 and to provide an update to the animal genetics community regarding the new usage of MeSH Bioconductor packages.

## Results

### Improvement in MeSH annotations

A suite of MeSH analysis Bioconductor packages has been closely maintained and updated at least once a year to keep up with the latest biomedical literature, including animal genetics. Table 1 compares the total number of genes annotated by MeSH terms between pre‐Bioconductor 3.14 and Bioconductor 3.14 for cattle, swine and chicken. The number of MeSH annotated genes was obtained from the previous literature.^2^,^3^ An improvement in MeSH annotations was observed for all three species, as the numbers of annotated genes increased by 127, 50 and 23% for cattle, swine and chicken respectively. This suggests that the findings reported in inferential studies (e.g. GWAS and RNA‐seq analysis) were successfully incorporated in MeSH annotations. Because genes of farm animals are progressively being annotated in MeSH, downstream MeSH enrichment analysis is a powerful approach for aiding biological interpretations as well as generating biological hypotheses.

**Table 1:** Total number of genes annotated with MeSH in cattle, swine and chicken

Species	Pre‐Bioconductor 3.14	Bioconductor 3.14
Cattle	17 310^1^	39 273
Swine	15 786^1^	23 672
Chicken	9043^2^	11 133

^1^ Bioconductor 3.0.

^2^ Bioconductor 3.2.

### New MeSH enrichment analysis framework

Previously, the responsibility of installing Bioconductor annotation packages that mapped MeSH IDs and Entrez Gene IDs for each species to perform enrichment analysis (e.g. MeSH.Bta.eg.db for cattle) lay with the end user. Bioconductor 3.14 saw a major change, where instead of downloading and installing these packages, all annotation data are now stored in and can be accessed from a cloud server via the AnnotationHub Bioconductor package.^4^ One of the primary reasons behind this change is data reproducibility, a critical aspect of scientific research. Cloud storage allows developers and users to simultaneously maintain different versions of their data, thereby making provisions for old data to be archived. Additionally, the AnnotationHub style allows ease of practice during package installation by reducing the Bioconductor packages that need to be installed. The BiocFileCache Bioconductor package additionally supports the feature wherein a dataset once downloaded from AnnotationHub is stored as a cache file and is not retrieved from the server again. Implementation of such modern features in recent Bioconductor saves time otherwise spent on downloading packages and data. The R code for MeSH enrichment analysis and creation of farm animal annotation data under the new AnnotationHub framework is available in Appendix S1 and in a reproducible manner on Docker Hub (Appendix S2).

## Supporting information


**Appendix S1**. R code to perform MeSH enrichment analysis and to create farm animal annotation data under the AnnotationHub framework.

**Appendix S2**. Instructions on running the MeSH docker image available on Docker Hub.
