# Supplementary material for: Improved MeSH analysis software tools for farm animals
Source: Anim Genet. 2021 Dec 2;53(1):171–2. doi: 10.1111/age.13159 (PMC9300174; doi:10.1111/age.13159)
Supplement: Supplementary file 1 — Appendix S1. R code to perform MeSH enrichment analysis and to create farm animal annotation data under the AnnotationHub framework. [file AGE-53-171-s001.pdf]

MeSH Analysis Tutorial

Sabrina Amorim, Koki Tsuyuzaki, Itoshi Nikaido and Gota Morota

November, 2021

Setting

```
# Package Install
if (!requireNamespace("BiocManager", quietly = TRUE)){
    install.packages("BiocManager")
}

pkgs <- c("knitr",  "rmdformats", "biomaRt", "AnnotationHub",
"meshr", "MeSHDbi", "readxl", "tagcloud", "RColorBrewer")
for(i in seq_along(pkgs)){
    if (!requireNamespace(pkgs[i], quietly = TRUE)){
        BiocManager::install(pkgs[i], suppressUpdates=TRUE, force=TRUE)
    }
}

# Package Loading
library("knitr")
library("rmdformats")
library("biomaRt")
library("AnnotationHub")
library("meshr")
library("MeSHDbi")
library("readxl")
library("tagcloud")
library("RColorBrewer")
```

Why use MeSH?

Medical Subject Headings, also known as MeSH, is a comprehensive collection of biological annotations annually updated by the National Library of Medicine. The collection includes more than **27,000** biological terms for many species, including crops and farm animals (Tsuyuzaki et al. 2015; Morota et al. 2015, 2016; Beissinger and Morota, 2017). The MeSH software packages (Tsuyuzaki et al. 2015) have been successfully applied to **swine, cattle, horses, chicken, and turkey** (Morota et al. 2015, 2016; Júnior et al. 2017; Abdalla et al. 2020). These studies demonstrated the utility of MeSH in improving the biological interpretation of gene sets and understanding the genetic architecture of complex traits in farm animals. The R code below shows how to perform MeSH enrichment analysis in Bioconductor 3.14 or later. The major change made in Bioconductor 3.14 is shown in Figure 1.

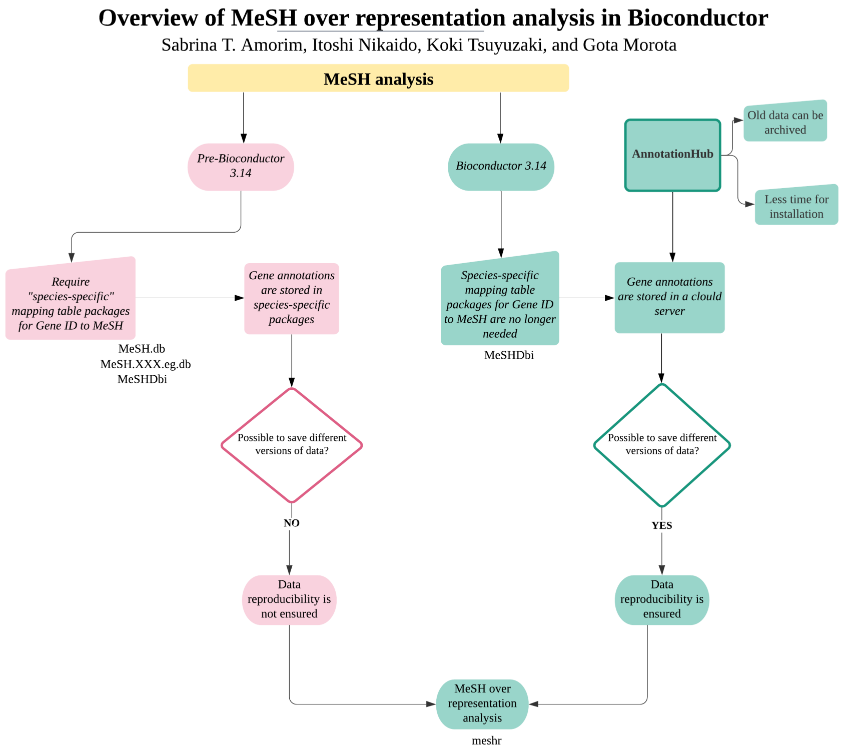

Create a vector of background genes

We first create a vector of background genes.

```
## Access to biomaRt
mart <- useMart(biomart = "ENSEMBL_MART_ENSEMBL", dataset = "btaurus_gene_ensembl",
                host="uswest.ensembl.org")
univ.geneID <- getBM(attributes=c("ensembl_gene_id", "entrezgene_id",
                                  "hgnc_symbol"), mart = mart) # 27962
## Remove genes with no corresponding Entrez Gene ID
univ.geneID2 <- univ.geneID[!is.na(univ.geneID[,2]),] # 21122
## Remove duplicated Entrez Gene ID
univ.geneID3 <- univ.geneID2[ !duplicated(univ.geneID2[,2]),] # 20946
```

Note that the above code can be easily switched to any species by changing the “btaurus_gene_ensembl” parameter in the **useMart** function.

Download and read input file

This tutorial will use data from an RNA-seq study by Li et al. (2015). The authors reported a list of differentially expressed genes in the ruminal wall of grass-fed and grain-fed Angus cattle. Please download the file S3 Table from the journal website.

```r
## Read data
my.geneID <- read_excel("pone.0116437.s004.xls", col_names = TRUE, sheet = 1, na = "NA") # 342
## Change the 1st column name to "ensembl_gene_id".
colnames(my.geneID)[1] <- "ensembl_gene_id"

## Merge two files
my.geneID2 <- merge(my.geneID, univ.geneID3, by = "ensembl_gene_id") # 307
## Remove duplicated Entrez Gene ID
my.geneID3 <- my.geneID2[!duplicated(my.geneID2$entrezgene), ] # 307
```

Access to MeSH data on AnnotationHub

MeSH terms are designated by articles indexed in PubMed by the National Library of Medicine and can be directly mapped to genes . There are currently **19** categories.

We will pay particular attention to the following four MeSH categories. **A** (Anatomy), **C** (Diseases), **D** (Chemicals and Drugs), **G** (Phenomena and Process).

The usage of the MeSH enrichment analysis framework has changed in Bioconductor 3.14. Species-specific annotation packages are no longer distributed. Instead, the MeSH annotation data are now stored in a cloud accessible via the *AnnotationHub* package. , This change ensures data reproducibility (e.g., the user can specify versions of the data, such as *"v002"*).

The first step is to download MeSH annotated genes for *Bos taurus* from the cloud. We will first download MeSH annotations using the **AnnotationHub** package, which returns the metadata of all the data stored in the AnnotationHub server.

```r
ah <- AnnotationHub()

dbfile1 <- query(ah, c("MeSHDb", "MeSH.db", "v002"))[[1]]
MeSH.db <- MeSHDbi::MeSHDb(dbfile1)
```

Creating the Cattle dataset - MeSH.Bta.eg.db

```r
dbfile2 <- query(ah, c("MeSHDb", "Bos taurus", "v002"))[[1]]
MeSH.Bta.eg.db <- MeSHDbi::MeSHDb(dbfile2) # Converting SQLite file into MESHDb object
MeSH.Bta.eg.db
```

```
[1] "##### class ####"
[1] "MeSHDb"
attr(,"package")
[1] "MeSHDbi"
[1] "##### connection #####"
<SQLiteConnection>
  Path: /Users/morota/Library/Caches/org.R-project.R/R/AnnotationHub/217b03e5fe_104593
  Extensions: TRUE
[1] "##### sqlite file #####"
                                                                    AH97847
"/Users/morota/Library/Caches/org.R-project.R/R/AnnotationHub/217b03e5fe_104593"
```

Note that the above code can be easily switched to any species by changing the "*Bos taurus*" parameter in the **query** function.

MeSH Enrichment Analysis

MeSH enrichment analysis can be performed using the *meshr* package. Let's perform MeSH enrichment analysis for **Chemicals and Drugs** category by setting **category="D"**.

```
# Chemicals and Drugs
meshParams.D <- new("MeSHHyperGParams",
                    geneIds = my.geneID3[, 6],
                    universeGeneIds = univ.geneID3[, 2],
                    annotation = "MeSH.Bta.eg.db",# Match between MeSH IDs and Entrez Gene IDs
                    meshdb = "MeSH.db",
                    category = "D",
                    database = "gene2pubmed", # data from PubMed
                    pvalueCutoff = 0.5, pAdjust = "none")
meshR.D <- meshHyperGTest(meshParams.D)

head(summary(meshR.D)[!duplicated(summary(meshR.D)[,7]), ]) # Show the first 6 MeSH terms
```

```
       MESHID       Pvalue  OddsRatio   ExpCount Count Size
40144 D019765 3.118305e-06        Inf 0.04397021     3    3
30966 D015815 5.163257e-06  12.016083 0.68886661     7   47
5392  D003871 3.050802e-05 101.827303 0.07328368     3    5
51692 D058575 5.437436e-05  24.756076 0.21985105     4   15
49644 D051182 6.035269e-05  67.881579 0.08794042     3    6
5265  D003598 2.015881e-04   6.397689 1.20185238     7   82
                    MESHTERM GENEID SOURCEID
40144   Chondroitin ABC Lyase 282654 11322944
30966 Cell Adhesion Molecules 505775 30385793
5392         Dermatan Sulfate 280760  6654908
51692                 Decorin 280760 25012297
49644             Desmogleins 281131  1702628
5265    Cytoskeletal Proteins 281597 11018051
```

The user can easily change a MeSH category by changing the **category** parameter. Let's perform enrichment analysis for **Diseases** category by changing "**D**" for "**C**".

```
meshParams.C <- new("MeSHHyperGParams",
                    geneIds = my.geneID3[, 6],
                    universeGeneIds = univ.geneID3[, 2],
                    annotation = "MeSH.Bta.eg.db",
                    meshdb = "MeSH.db",
                    category = "C",
                    database = "gene2pubmed",
                    pvalueCutoff = 0.5, pAdjust = "none")

meshR.C <- meshHyperGTest(meshParams.C)

head(summary(meshR.C)[!duplicated(summary(meshR.C)[,7]), ]) # Show the first 6 MeSH terms
```

```
      MESHID       Pvalue  OddsRatio   ExpCount Count Size
2358 D006566 0.0000926359  20.945418 0.24916452     4   17
2258 D005355 0.0006361546 135.331148 0.04397021     2    3
2201 D003048 0.0072016031  19.327400 0.13191063     2    9
2419 D008414 0.0132859414   3.245214 1.93468920     6  132
2200 D002534 0.0146567364        Inf 0.01465674     1    1
2244 D004802 0.0146567364        Inf 0.01465674     1    1
```

```
                      MESHTERM GENEID SOURCEID
2358 Herpesviridae Infections 281496 22823939
2258                 Fibrosis 280760  8907183
2201              Coccidiosis 337897 26936628
2419         Mastitis, Bovine 281345 25946326
2200           Hypoxia, Brain 282369 12716435
2244             Eosinophilia 404072 15916812
```

MeSH enrichment analysis for Anatomy (category = **"A"**).

```
meshParams.A <- new("MeSHHyperGParams",
                    geneIds = my.geneID3[, 6],

                    universeGeneIds = univ.geneID3[, 2],
                    annotation = "MeSH.Bta.eg.db",
                    meshdb = "MeSH.db",
                    category = "A",
                    database = "gene2pubmed",
                    pvalueCutoff = 0.5, pAdjust = "none")
meshR.A <- meshHyperGTest(meshParams.A)

head(summary(meshR.A)[!duplicated(summary(meshR.A)[,7]), ]) # Show the first 6 MeSH terms
```

```
       MESHID       Pvalue OddsRatio    ExpCount Count  Size        MESHTERM
17365 D014998 6.907907e-08  2.452987 239.3591617   275 16331    Y Chromosome
2749  D002478 9.472117e-05  2.312057  13.4841975    29   920 Cells, Cultured
5901  D003896 1.653370e-04 40.725000   0.1172539     3     8      Desmosomes
1055  D002460 1.724265e-04  2.478690   9.9079538    23   676       Cell Line
6345  D004717 3.732997e-04  4.948145   1.7441516     8   119     Endometrium
11140 D008214 9.180794e-04 10.466108   0.4397021     4    30     Lymphocytes
         GENEID SOURCEID
17365    522449 19393038
2749     444881 26499291
5901     281131  1706270
1055     286811 29543378
6345  100313957 28605413
11140    282530 19630880
```

MeSH enrichment analysis for Phenomena and Processes (category = **"G"**).

```
meshParams.G <- new("MeSHHyperGParams",
                    geneIds = my.geneID3[, 6],
                    universeGeneIds = univ.geneID3[, 2],
                    annotation = "MeSH.Bta.eg.db",
                    meshdb = "MeSH.db",
                    category = "G",
                    database = "gene2pubmed",
                    pvalueCutoff = 0.5, pAdjust = "none")
meshR.G <- meshHyperGTest(meshParams.G)

head(summary(meshR.G)[!duplicated(summary(meshR.G)[,7]), ]) # Show the first 6 MeSH terms
```

```
        MESHID       Pvalue OddsRatio    ExpCount Count  Size
122349 D016678 3.675469e-08  2.550414 241.0886565   277 16449
73275  D014998 6.907907e-08  2.452987 239.3591617   275 16331
101019 D015894 6.978741e-08  2.452299 239.3738184   275 16332
```

```
179294 D026801 6.978741e-08  2.452299 239.3738184   275 16332
38392  D008285 1.177329e-04 19.448373   0.2638213     4    18
1489   D000595 1.281753e-04  1.832364  31.7025208    53  2163
                                  MESHTERM GENEID SOURCEID
122349                              Genome 515523 19393038
73275                         Y Chromosome 511375 19393038
101019                      Genome, Human 618052 19393038
179294                             Synteny 507340 19393038
38392  Major Histocompatibility Complex 282535  7611993
1489                   Amino Acid Sequence 281951 21029378
```

Visualization for MeSH Enrichment Analysis

The *tagcloud* function from the tagcloud package creates a word cloud. In our case, the size of the MeSH terms is proportional to their extent of enrichment.

Create a Word Cloud for Chemicals and Drugs category.

```
meshR <- meshR.D # Use your meshR.D results from the Mesh-ORA analysis
meshR.summary <- summary(meshR)[!duplicated(summary(meshR)[, 7]), ] # Extract unique elements
tags <- strmultline(meshR.summary$MESHTERM)
weights <- -log(meshR.summary$Pvalue)
or <- meshR.summary$OddsRatio
colors <- smoothPalette(weights, pal = brewer.pal(11, "Spectral"))
tagcloud(tags, weights = weights, col = colors, order = "size")
```

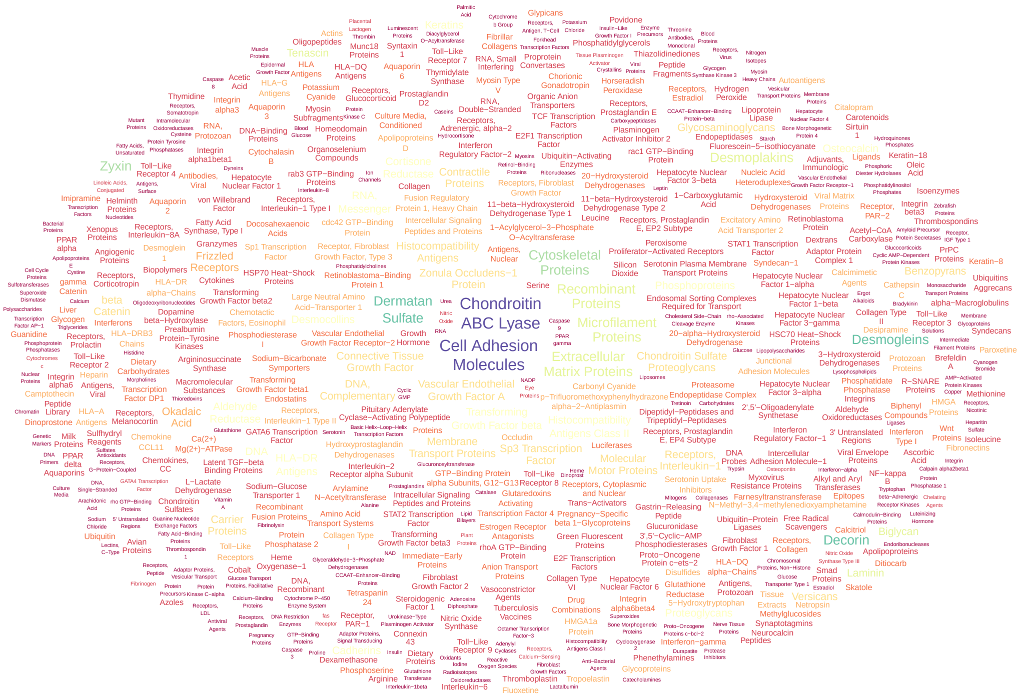

Note: This is a suggested color palette. The user is free to choose any color palette. More options can be found here.

```
# Diseases
meshR <- meshR.C
```

```
meshR.summary <- summary(meshR)[!duplicated(summary(meshR)[, 7]), ]
tags <- strmultline(meshR.summary$MESHTERM)
weights <- -log(meshR.summary$Pvalue)
or <- meshR.summary$OddsRatio
colors <- smoothPalette(weights, pal = brewer.pal(11, "Spectral"))
tagcloud(tags, weights = weights, col = colors, order = "size")
```

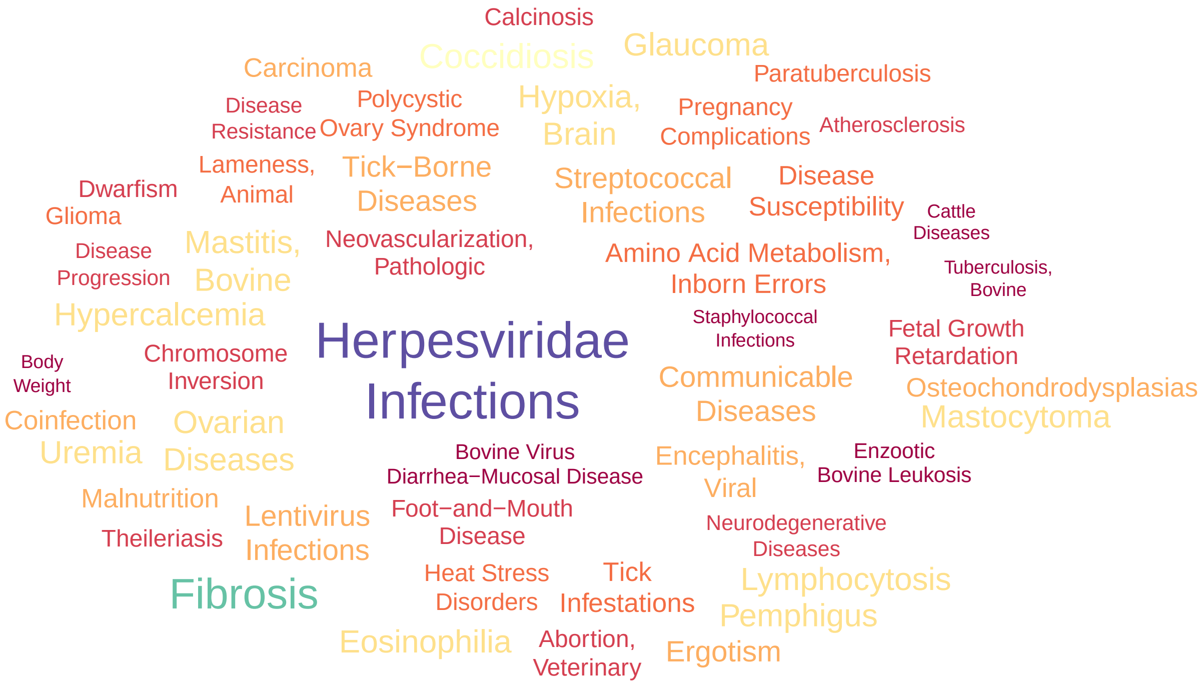


```
# Anatomy
meshR <- meshR.A
meshR.summary <- summary(meshR)[!duplicated(summary(meshR)[, 7]), ]
tags <- strmultline(meshR.summary$MESHTERM)
weights <- -log(meshR.summary$Pvalue)
or <- meshR.summary$OddsRatio
colors <- smoothPalette(weights, pal = brewer.pal(11, "Spectral"))
tagcloud(tags, weights = weights, col = colors, order = "size")
```

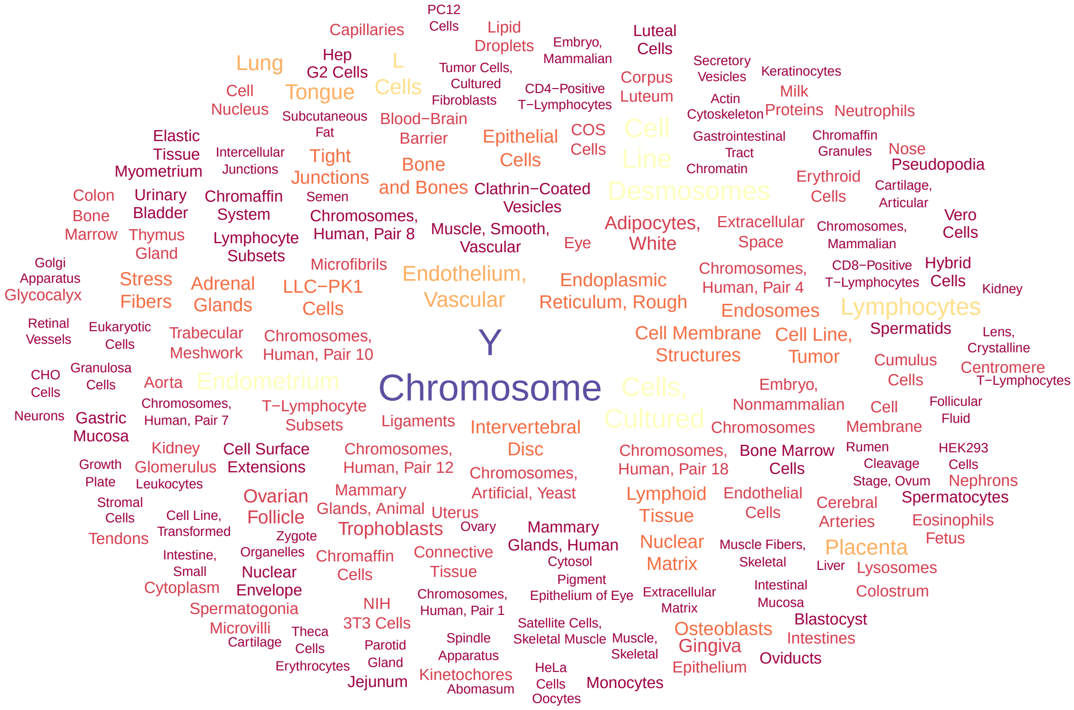


```r
# Phenomena and Processes
meshR <- meshR.G
meshR.summary <- summary(meshR)[!duplicated(summary(meshR)[, 7]), ]
tags <- strmultline(meshR.summary$MESHTERM)
weights <- -log(meshR.summary$Pvalue)
or <- meshR.summary$OddsRatio
colors <- smoothPalette(weights, pal = brewer.pal(11, "Spectral"))
tagcloud(tags, weights = weights, col = colors, order = "size")
```

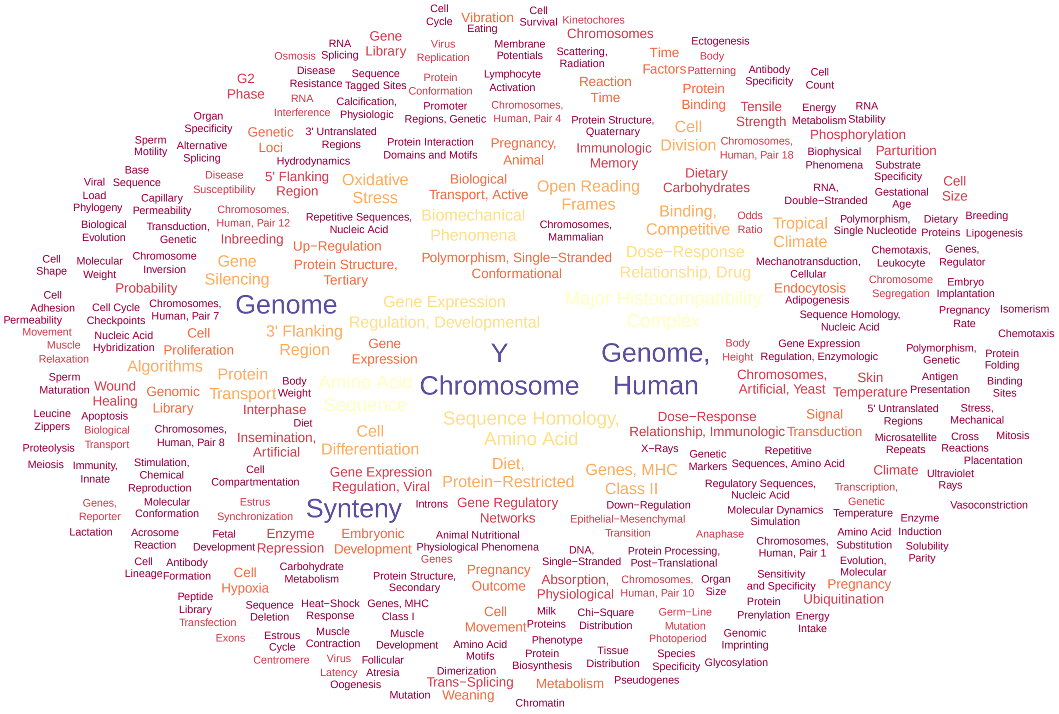

Other farm animals species

MeSH annotations for other species can be easily downloaded by providing the species names. The following code downloads the MeSH annotation databases for **Swine** and **Chicken**.

Swine - MeSH.Ssc.eg.db

```
dbfile3 <- query(ah, c("MeSHDb", "Sus scrofa", "v002"))[[1]]
MeSH.Ssc.eg.db <- MeSHDbi::MeSHDb(dbfile3)
MeSH.Ssc.eg.db
```

```
[1] "##### class ####"
[1] "MeSHDb"
attr(,"package")
[1] "MeSHDbi"
[1] "##### connection #####"
<SQLiteConnection>
  Path: /Users/morota/Library/Caches/org.R-project.R/R/AnnotationHub/59b825b7bf58_104648
  Extensions: TRUE
[1] "##### sqlite file #####"
                                                                                 AH97902
"/Users/morota/Library/Caches/org.R-project.R/R/AnnotationHub/59b825b7bf58_104648"
```

Chicken - MeSH.Gga.eg.db

```
dbfile4 <- query(ah, c("MeSHDb", "Gallus gallus", "v002"))[[1]]
MeSH.Gga.eg.db <- MeSHDbi::MeSHDb(dbfile4)
MeSH.Gga.eg.db
```

```
[1] "##### class ####"
[1] "MeSHDb"
attr(,"package")
[1] "MeSHDbi"
[1] "##### connection #####"
<SQLiteConnection>
  Path: /Users/morota/Library/Caches/org.R-project.R/R/AnnotationHub/59b81bdb2a55_104619
  Extensions: TRUE
[1] "##### sqlite file #####"
                                                                                 AH97873
"/Users/morota/Library/Caches/org.R-project.R/R/AnnotationHub/59b81bdb2a55_104619"
```

Final remark

The MeSH database is updated frequently to reflect the findings from newly published papers in animal genetics, and we provide the database biannually along with Bioconductor updates. MeSH enrichment analysis may help to provide new insights into the biological interpretation of genes influencing complex traits in livestock species. The above R code is also available at https://sabrinaam.github.io/.
