# Supplementary material for: Improved MeSH analysis software tools for farm animals
Source: Anim Genet. 2021 Dec 2;53(1):171–2. doi: 10.1111/age.13159 (PMC9300174; doi:10.1111/age.13159)
Supplement: Supplementary file 2 — Appendix S2. Instructions on running the MeSH docker image available on Docker Hub. [file AGE-53-171-s002.pdf]

Supporting file: Improved MeSH analysis software tools for farm animals

Sabrina T. Amorim[1], Koki Tsuyuzaki[2], Itoshi Nikaido[2], and Gota Morota[1*]

[1]Department of Animal and Poultry Sciences, Virginia Polytechnic Institute and State University, Blacksburg, VA, 24061, USA.
[2]Laboratory for Bioinformatics Research, RIKEN Center for Biosystems Dynamics Research, 2-1 Hirosawa, Wako, Saitama, 351-0198, Japan.

[*] Corresponding author
E-mail: morota@vt.edu (GM)

ORCID: 0000-0003-4130-2040 (STA), 0000-0003-3797-2148 (KT), 0000-0002-7261-2570 (IN), and 0000-0002-3567-6911 (GM)

Email addresses: asabrina@vt.edu (STA), koki.tsuyuzaki@gmail.com (KT), itoshi.nikaido@riken.jp (IN), and morota@vt.edu (GM).

Instructions on running the MeSH docker image available on Docker Hub

This supporting file shows a step-by-step guide on running the MeSH docker image available on Docker Hub (`https://hub.docker.com/`). Docker enables building, running, and managing containers on the cloud, allowing anyone to run the code in any environment.

Step 1

Install Docker Desktop for your machine's operating system (e.g., Mac, Windows, or Linux) at `https://docs.docker.com/get-docker/` if not installed on your computer yet.

Step 2

Start Docker Desktop so that you can use docker commands in the command line.

Step 3

Run the following docker command in the command line. It is required to set a password.

```
docker run -e PASSWORD=your_password_here
    -p 8787:8787 koki/rstudio_mesh
```

Step 4

Visit http://localhost:8787 in your browser and log in with username *rstudio* and the password you set.

```
http://localhost:8787/
```

Step 5

You will find an Rmd file for HTML output. Double-click on the file to open it. Click the "run" button at each chunk in the Rmd file one by one to see how the MeSH analysis works. Alternatively, you can click the "Knit" button to run all the chunks at once.
